# DSD – An integrated, web-accessible database of Dehydrogenase Enzyme Stereospecificities

**DOI:** 10.1186/1471-2105-6-283

**Published:** 2005-11-30

**Authors:** Christopher P Toseland, Helen M McSparron, Darren R Flower

**Affiliations:** 1 The Jenner Institute, University of Oxford, Compton, Berkshire, RG20 7NN, UK

## Abstract

**Background:**

Dehydrogenase enzymes belong to the oxidoreductase class and utilise the coenzymes NAD and NADP. Stereo-selectivity is focused on the C4 hydrogen atoms of the nicotinamide ring of NAD(P). Depending upon which hydrogen is transferred at the C4 location, the enzyme is designated as A or B stereospecific.

**Description:**

The Dehydrogenase Stereospecificity Database *v1.0 *(DSD) provides a compilation of enzyme stereochemical data, as sourced from the primary literature, in the form of a web-accessible database. There are two search engines, a menu driven search and a BLAST search. The entries are also linked to several external databases, including the NCBI and the Protein Data Bank, providing wide background information. The database is freely available online at:

**Conclusion:**

DSD is a unique compilation available on-line for the first time which provides a key resource for the comparative analysis of reductase hydrogen transfer stereospecificity. As databases increasingly form the backbone of science, largely complete databases such as DSD, are a vital addition.

## Background

**Figure 1:**
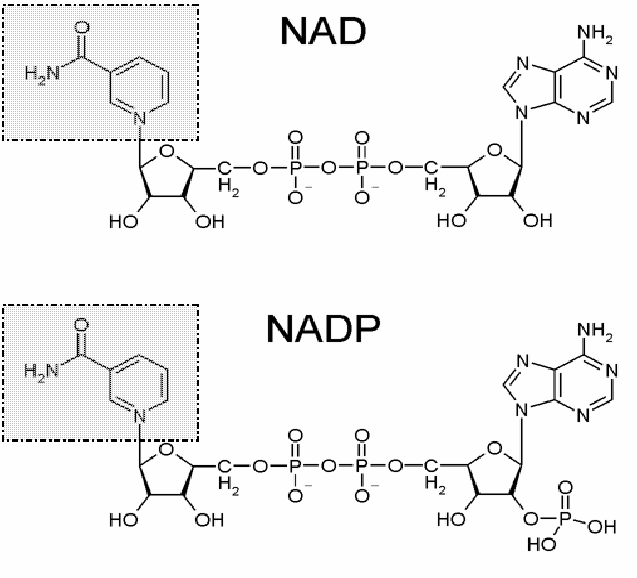
**The structures of pyridine nucleotide coenzymes, NAD and NADP. **The nicotinamide rings which are pivotal to the hydride transfer, are highlighted. The rings are bound to ribose, which with respect to NAD, is linked to another ribose sugar and adenine through two phosphate groups. With regard to NADP, the ribose and nicotinamide ring are bound by two phosphate groups to adenosine monophosphate.

Dehydrogenase enzymes exhibit stereo-selectivity for diastereotopic hydrogen atoms, situated at the C4 position of the dihydronicotinamide ring of the pyridine nucleotide coenzymes, Nicotinamide Adenine Dinucleotide (NAD) and Nicotinamide Adenine Dinucleotide Phosphate (NADP). The dehydrogenases belong to the oxidoreductase group of enzymes (E.C. Class 1), which oxidise their substrates by transferring hydrogen from NAD or NADP. These molecules act as high energy electron donors, plus low energy acceptors and are derived from the vitamin niacin; the molecules consist of two ribose groups, linked by two phosphates, which are in turn bonded to two bases, adenine and nicotinamide (Figure 1a). NADP is analogous to NAD, but NADP contains an additional phosphate group which forms an adenosine monophosphate group. The whole of the coenzyme is involved in interactions with the enzyme, but hydrogen transfer occurs only between substrate and the nicotinamide ring; other parts of the coenzyme are solely involved in binding to the enzyme and not in hydrogen transfer. NAD(P) are ubiquitous in all living systems and are implicated in a vast number of reactions involving almost 20% of classified enzymes [1].

The structure of NAD(P) allows presentation of either hydrogen depending on the orientation of the nicotinamide ring, which can be bound in either *syn*- or *anti*- conformations, which are related by a 180 degree rotation about the glycosidic bond linking the nicotinamide base to the C1 of ribose [2] (Figure 1b). There have been several designations for the C4 hydrogens since the 1950's, however the approved systems are A/B [3] and *pro R*/*pro S *[1]. The *pro R*/*pro S *system is superior to A/B as it denotes the absolute configuration of the atom. The A/B system works by comparing enzyme stereo-selectivity with that of yeast alcohol dehydrogenase, which is an A stereospecific enzyme. Enzymes with the same stereospecificity are designated as A, while other enzymes are denoted as B stereospecific [3]. For comparison between the two systems, A is essentially the same as *pro R *and B is the same as *pro S *[4].

Fisher [5] was the first to observe these stereospecific reactions. It was shown that yeast alcohol dehydrogenase catalysed the hydride transfer between the substrate and NAD. Further studies were able to show that such stereospecificity was not unique to alcohol dehydrogenases; the same hydride transfer was seen in malate [6] and lactate [7] dehydrogenases. However, it was not until 1955 that B stereospecific enzymes were discovered: β-hydroxysteroid dehydrogenase [8] and transhydrogenase [9]. These initial investigations were conducted using mass spectroscopy, with deuterium-labelled coenzymes [10]. Tritium-labelled coenzymes were later used with scintillation counting [11,12]. The C4 location of the nicotinamide ring is labelled and the isotope level determined [13]. Both of these techniques were not adequate for large-scale studies, as they are laborious, time-consuming and require multiple purification steps [12]. In 1976, Arnold [14] introduced a technique based on ^1^H Nuclear Magnetic Resonance (NMR) spectroscopy. This method was accurate and allowed direct measurement of deuterium content at the reaction site, as well as being safer and more facile than previous techniques.

The determination of stereospecificity is essential for gaining an understanding of the reaction mechanism of an enzyme. Most important of all, is the fact that knowledge of the stereochemistry based on hydride transfer can provide an insight into the relative substrate and coenzyme binding orientations; this can be instrumental in designing strategies to control and regulate reactions [13]. We have therefore accumulated a comprehensive compendium of hydride transfer stereospecificity data, and placed the information in a searchable database. The Dehydrogenase Stereospecificity Database (DSD) *v1.0 *database designates enzymes as either A or B stereospecific, for a given coenzyme. Two decades ago, You [1] compiled available stereospecificity data in a landmark review; we have used this and added experimental data determined during the past 20 years, to bring the compilation up to date. This provides a unique and accurate collection of dehydrogenase enzyme stereospecificities.

## Construction and content

### Database design and development

**Figure 2:**
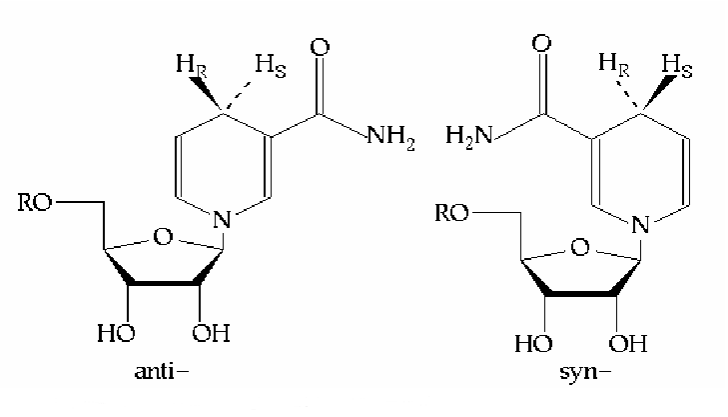
**The *anti*- and *syn*- conformations of the nicotinamide ring. **A stereospecific enzymes will bind NAD(P) in the *anti*- conformation, which B stereospecific enzymes bind NAD(P) in the *syn*- conformation.

DSD *v1.0 *has been implemented as a postgreSQL relational database. This establishes a flexible infrastructure capable of addressing all foreseeable developments of the archive. The data was initially compiled in a Microsoft ACCESS database after addition of data from previous studies and exhaustive searching of the primary literature. The postgreSQL database is structured into seven normalised tables, populated from a flat-file export of the ACCESS database using PERL scripts integrated with SQL. See Figure 2. As data are continually accumulating, archiving data is an on-going process: automatic, periodic updates will be made to the postgreSQL database. The DSD user interface is a series of HTML pages. Searchable HTML forms are available within the DSD site, and provide either a detailed DSD search or a DSD BLAST search. These forms target either a PERL/SQL script or a CGI script. These will in turn query the database. The bespoke search engine facilitates fast, efficient and flexible data retrieval (see 'Searching the Database'). DSD is freely available on the web .

### Implementing the database

The data was sourced from a previous compilation [1] and exhaustive searching of the primary literature. The archive consists of entries containing the enzyme name, the species from which it was derived, experimental technique, coenzymes and the stereospecificity. For simplicity, DSD utilises the A/B naming system [3], which was adopted by You [1].

Cross-references to key external databases are also made. These provide links to the protein sequence, using NCBI Entrez-Protein, and the relevant protein structure in the Protein Data Bank [15]. If applicable, the Enzyme Classification is given with links to the Enzyme Nomenclature and Classification database, developed in line with the International Union of Biochemistry and Molecular Biology, providing details of the enzyme reactions. In addition, a full literature reference is given with links to the NCBI PubMed journals database. These links provide the user with necessary background knowledge for the archived enzyme. A full description of the entry fields is given in Table 1.

**Table 1:** Contents of the database entries.

Entry Field	Description
Enzyme	The relevant protein and provides a link to the NCBI Entrez-Protein sequence
PDB	PDB identification code, plus a link to the equivalent structure
EC	The Enzymes Commissions identification number, plus a link to the external database
Source Species	Species in which the protein is found
Coenzyme	The coenzyme used in the experimental determination e.g. NAD(P)
Stereospecificity	Given according to the A/B nomenclature
Method	Experiment techniques used to obtain data e.g. NMR
Temperature	Temperature at which the experiment was carried out
pH	Range or fixed pH at which the experiment were carried out
Conditions	Concentrations, etc used in the experiment
Reference	Full literature reference with link to the PubMed database

The usefulness of a database is governed by the accuracy of the data it contains. The data contained in DSD *v1.0 *was compiled manually from previously published, peer-reviewed articles, and verified, where possible, from the original literature. This suggests that, compared to some other databases, DSD will be accurate and reliable. Moreover, to maintain this accuracy, data was only added from the primary literature for widely accepted experimental techniques, such as NMR spectroscopy. Experimental conditions have also been included, with 113 temperature measurements, and 117 pH values, and 135 concentration measurements. As logistical considerations preclude us from undertaking independent verification of the data, we are obliged to trust the data reported in the literature.

### Searching the database

**Figure 3:**
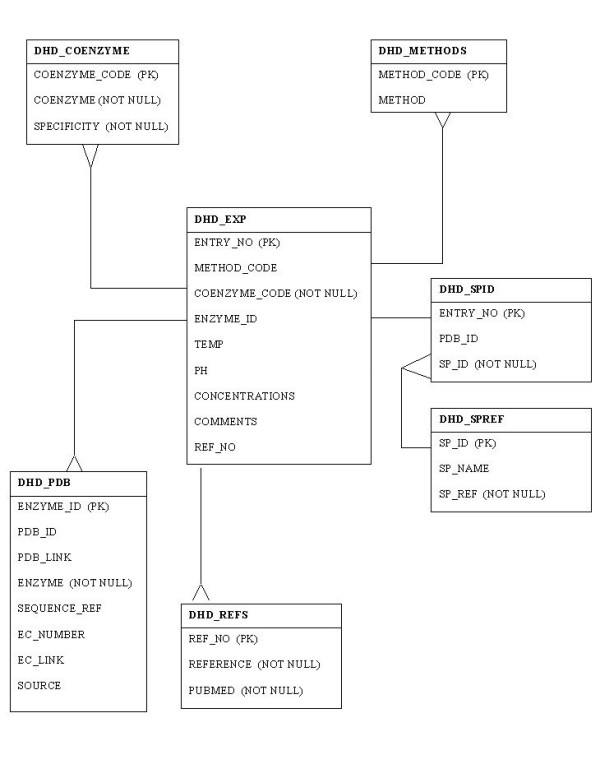
DSD Table Schema.

Two search methods for DSD are available: a menu-based interface and an interface based on Basic Local Alignment Search Tool (BLAST) [16]. The implementation of a menu-based bespoke search system allows the user to perform either a broad or a detailed search from one simple search interface. The database is searched by selecting enzyme, source or experimental method, or a combination thereof. The HTML form passes the search criteria to SQL database queries. The data returned is formatted using HTML before being printed to the screen. The initial results page summarises the data fields 'PDB', 'Enzyme', 'Source', 'Method' and 'EC Number' for all hits. A 'View Data' button is available to obtain experimental detail for the selected data set. See Figure 3.

**Figure 4:**
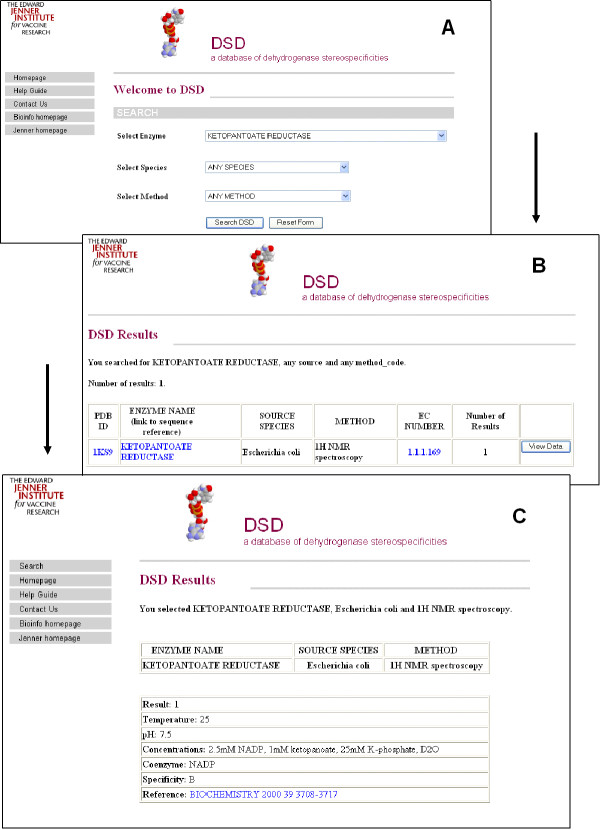
**Overview of the DSD search**. The search menus (**A**) provide an easy method of selecting for one, or all of, the three categories. The search will display a list of matches for the criteria (**B**), the DSD entry can then be viewed for the selected enzyme (**C**).

The alternative search interface is based on BLAST [16]. A local database of protein sequences found in DSD was compiled from SWISS-PROT [17] and an additional postgreSQL table was created to hold this data. The local database is searched using the NCBI BLASTP and BLASTX programs [16], allowing for input of protein or nucleotide sequences. The HTML Front-End connects to a web server-based PL/CGI scripts which interacts with the BLASTP or BLASTX programs. In the output, DSD entries are linked to SWISS-PROT [17] using accession codes.

## Utility and discussion

### Implementation and initial analysis

DSD is a relatively small database but is, nonetheless, essentially complete. The current size of the database is 461 entries, which corresponds to 185 distinct enzymes from 121 different source species. From the current data, we can conclude that NAD is the more frequently used coenzyme, since there are 272 entries for enzymes using NAD and 189 using NADP, which represents a 60:40 divide between the two main pyridine nucleotide coenzymes NAD and NADP, respectively. This may simply reflect experimental bias and not any natural selection for a particular coenzyme. It has been reported several times before that there is an approximately equal distribution of A and B stereospecific dehydrogenase enzymes [1,13] and that none are stereo-random; the up-to-date data contained within DSD *v1.0 *is consistent with this view. Our data displays an approximately even distribution, with 236 occurrences of A stereospecific enzymes and 225 occurrences of B stereospecific enzymes (Table 2).

**Table 2:** Overview of the DSD data.

**Coenzyme**	**Frequency**
**NAD**	**271**
**NADP**	**181**
	
**Stereospecificity**	**Occurrence**


**A**	**242**
**B**	**232**

The distribution of coenzymes can be seen above: a 60:40 division is present between the NAD and NADP, respectively. The overall distribution between the stereospecificities of dehydrogenase enzymes is as observed previously. Our data is analogous to prior studies (You, 1985; Hallis *et al*., 1998) which indicated an approximately equal distribution.

A-stereospecific enzymes may have evolved before B-stereospecific enzymes, since A-stereospecific enzyme substrates are smaller and less complex compounds than those of B-stereospecific enzymes [2]. Prebiotic chemical processes would favour simpler molecules, while primitive organisms would have had rudimentary catabolic systems able to derive energy from small molecules. Due to structural conservation, it is also possible to deduce evolutionary correlations between diverse catalysts by comparing the binding orientations of the coenzymes, substrate and enzyme active site [18]. On this basis, Benner [19,20] postulated that A stereospecific enzymes will bind NAD(P) with the ring in the *anti*-conformation, while B stereospecific enzymes will bind NAD(P) with the ring in the *syn*-conformation (Figure 1b). With respect to dehydrogenase enzymes, which have divergently evolved from a common precursor, the stereospecificity will be maintained for as long as the protein fold of the catalytic domain is conserved, for a given substrate [21]. An A to B transformation in stereospecificity, or *vice versa*, would require a total rearrangement of the essential functional residues, to allow the correct binding orientation. This is based on the structural constraints mentioned above and therefore such an exchange is inconceivable during divergent evolution [18]. Binding constraints may not be an overall determinant, as several groups of enzymes from different sources having contrasting stereospecificities [21].

A relationship between the stereospecificity and equilibrium constant of a hydride transfer has also been proposed: reactions with pKeq > 11.3 correlate with A stereospecificity and reactions with pKeq <11.3 correlate with B stereospecificity. However, as with many postulated generalisations, there are exceptions, such as 3α-hydroxysteroid dehydrogenase. The enzyme reaction has a pKeq ~ 8, within the B range yet it is A stereospecific [18]. This highlights the problems associated with forming generalisations about these reactions.

### Future work

With respect to future work, the database needs to be maintained and developed further, ensuring our links to external databases remain current and newly published data is added. Initially, as with all databases, random errors will have occurred due to human error during the data accumulation or will be extant within the original experimental data. The database will be assessed for errors and inconsistencies, thus maintaining, as far as possible, the overall veracity of our data. Moreover, feedback on the search interfaces and the general infrastructure will allow us to address issues raised and revise the database accordingly.

You [1] included data on several other pyridine nucleotide coenzymes, such as 3-acetylpyridine adenine Dinucleotide ((3AcPy)AD), 3-cyanopyridine adenine Dinucleotide ((CNPy)AD), thionicotinamide adenine dinucleotide ((TN)AD) and flavin adenine dinucleotide (FAD). We will seek to incorporate data on these and similar coenzymes in future versions of the database. Likewise, we will seek to enhance search capabilities within the database by including the ability to search for types of interaction sets of amino acids within user-defined distances of NAD(P) coenzymes. We will complement this with a simple visual summary.

## Conclusion

The DSD *v1.0 *database is a unique, up to date compilation of dehydrogenase stereospecificities, which has advanced on reviews put together over 20 years ago. We see the database as being relatively small, due to the constraints of information, but largely complete. As new data becomes available, the database will increase in size. The ease of access to the data is of great importance and the bespoke search system and the inclusion of a BLAST search greatly facilitates this. The addition of the cross-references to several external databases provides expansive background information. We hope the database will provide an important resource which will help enhance our understanding of enzyme mechanisms. In an age when databases are increasingly forming the backbone of science, largely complete databases, such as DSD, are an important addition.

## Availability and requirements

The database is available at  suitable for most graphical web browser.

## Authors' contributions

CPT compiled the database and developed the BLAST search tool. HM was responsible for developing all postGRESQL and perl code and also designed the HTML web front end. DRF originated the concept, designed the database, and identified relevant data sources. All authors have read and approved the final manuscript.
